# Semi-nested polymerase chain reaction-based detection of *Babesia* spp. in small ruminants from Northwest of Iran

**DOI:** 10.14202/vetworld.2018.268-273

**Published:** 2018-03-03

**Authors:** Ahad Bazmani, Amir Abolhooshyar, Abbas Imani-Baran, Hamid Akbari

**Affiliations:** 1 Infectious and Tropical Diseases Research Center, Tabriz University of Medical Sciences, Tabriz, Iran; 2 Department of Pathobiology, Faculty of Veterinary Medicine, University of Tabriz, Tabriz, Iran; 3 Department of Pathobiology, Faculty of Veterinary Medicine, University of Tabriz, Tabriz, Iran; 4 Department of Clinical Sciences, Faculty of Veterinary Medicine, University of Tabriz, Tabriz, Iran

**Keywords:** *Babesia motasi*, *Babesia ovis*, Iran, semi-nested polymerase chain reaction, small ruminants

## Abstract

**Aim:**

The present study aimed to detect *Babesia ovis* and *Babesia motasi* in the blood samples of sheep and goats from Northwest of Iran by the semi-nested polymerase chain reaction (PCR) technique.

**Materials and Methods:**

A total of 166 whole blood samples (including 123 sheep and 43 goats) were collected. In the first stage, the PCR was performed to amplify a piece of 18S rRNA gene of *Babesia* and *Theileria* genera. Then, semi-nested PCR was carried out on all PCR products to differentiate *B. ovis* and *B. motasi*.

**Results:**

The PCR indicated that totally, 19 (11.44%) out of 166 samples were positive for *Babesia* or *Theileria* spp. The semi-nested PCR showed that 38 samples (22.89%) were positive only for *B. ovis*. No significant association was found between the infection rate of *B. ovis* and age, gender and species of animals.

**Conclusion:**

In the present study, there was no evidence for *B. motasi* infection in small ruminants from Northwest of Iran. Therefore, *B. ovis* was the main causative agent of ovine Babesiosis in this region.

## Introduction

In small ruminants, babesiosis is associated with intraerythrocytic protozoan parasites such as *Babesia ovis*, *Babesia motasi*, and *Babesia crassa* which is characterized more commonly by fever, anemia, hemoglobinuria, and icterus [1,2]. The most pathogenic species of *Babesia* in small ruminants is *B. ovis*. However, *B. motasi* has a moderate virulence [3]. Ixodid ticks including *Rhipicephalus* and *Haemaphysalis* are the main vector of *B. ovis* and *B. motasi*, respectively [2]. Ovine babesiosis is of considerable economic importance in tropical and subtropical areas [4]. Therefore, screening and control programs should be considered to reduce the negative impact of the disease on livestock farming. The identification of *Babesia* spp. is commonly performed by microscopic examination of Giemsa stained blood smears. However, its low sensitivity has limited the utilization of this method in the epidemiological investigations [5]. Serological procedures can be used for detecting subclinical infections of *Babesia* spp. However, these methods are not specific due to false positive and negative results and cross-reactivity with other species of *Babesia* [6].

The high sensitivity and specificity of molecular methods provide an accurate technique for differentiation of *Babesia* infections [7,8]. The prevalence of ovine babesiosis in Iran has been studied based on microscopic, serologic, and molecular approaches [1,9-12]. In the most of studies in Iran, *B. ovis* has been reported as the main causative agent of ovine babesiosis [1,11]. However, *B. motasi* was isolated in the blood samples of small ruminants in some studies in Iran [1,13]. Consequently, there is still an uncertainty regarding the causative agents of ovine babesiosis in small ruminants of Iran.

Therefore, the present study aimed to examine the presence of 18S rRNA gene of both *B. ovis* and *B. motasi* in the blood samples of sheep and goats by the semi-nested polymerase chain reaction (PCR) technique.

## Materials and Methods

### Ethical approval

The study protocol was approved by the Ethical Committee of University of Tabriz (UT/D-0621).

### Study area

**Figure-1:**
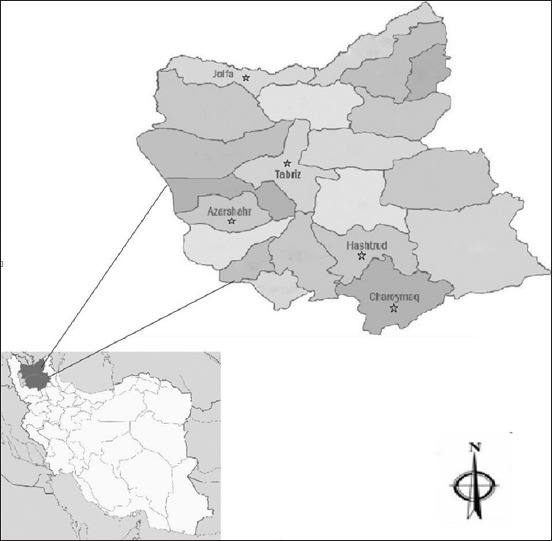
The map of East Azerbaijan Province of Iran. The blood samples were collected from areas which have been marked with a star.

This study was conducted in East Azerbaijan Province, Northwest of Iran during July 2015-September 2016 (Figure-1). The total area of this province is approximately 45650 Km^2^. The latitude and longitude are 36°54′-39º 26′ N and 45°7′-48°20′ E, respectively. The average annual rainfall, relative humidity and temperature are 300 mm, 44-67% and 12.3°C, respectively. The province has an arid to semi-arid climate. The sheep and goats farming is an important occupation in the rural areas of this province [14].

### Blood sampling

In the present study, the following formula was used to determine the appropriate sample size for detection of disease prevalence:


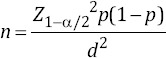


Where; n=sample size, *Z*_1−*α*/2_=1.96 for a confidence level of 95%, *p*=0.4 based on previous studies in Iran, Error of margin (*d*=0.08), therefore, *n*=144. A total of 166 blood samples were collected from the jugular vein of 123 sheep and 43 goats. Then, blood samples were filled in the 5 ml vacutainer tubes containing EDTA and kept on ice until transferred to the laboratory. During sampling, information about age, gender, and species of animals was recorded through a questionnaire.

### DNA extraction

The Genomic DNA of whole blood was extracted using a commercial kit (Pak Gene Yakhteh, Cat. No. PGEX2050) according to the manufacturer’s instruction.

### PCR and semi-nested assays

The PCR using an outer forward primer P1B-F: 5’-CACAGGGAGGTAGTGACAAG-3’ [15] and a reverse primer P2B-R: 5’-CTAAGAATTTCAC CTCTGACAGT-3’ [16] was performed to amplify a piece of 18S rRNA gene of *Babesia* and *Theileria* genera. The expected size of PCR products for *B. ovis* and *B. motasi* were 390 and 397 bp, respectively, which could not be differentiated in this stage. The PCR reaction was performed in a total volume of 20 μl containing 10× PCR buffer 2 µl, MgCl_2_ 2.5 mM, dNTP 0.2 mM, outer primers 0.2 pM of each (Bioneer Inc., Korea), Taq DNA polymerase 2U, template DNA 1.6 μl using the Astec PC818 thermal cycler (Astec, Japan). The thermal condition of PCR in this stage was 5 min incubation at 94°C for initial denaturation, 35 cycles of 40 s at 94°C, 40 s at 60°C, 60 s at 72°C and a final extension at 72°C for 7 min. The PCR products were electrophoresed on 1.5% agarose gel using Safe stain (CinnaGen Co., Iran).

The semi-nested PCR was performed on the primary PCR products to differentiate *Babesia* spp. A pair of primers, P3ov-F: 5′-GGCCTTTGCGTTACTTTGA-3′ (forward, AY260178 NCBI) [15] and P2B-R: 5’- CTAAGAATTTCACCTCTGACAGT-3’ (reverse) [16] were used to amplify a 178 bp fragment of the 18S rRNA gene of *B. ovis*. In addition, P4mot-F: 5′-CGCGATTCCGTTATTGGAG-3′ (forward, AY260179 NCBI) [5] and P2B-R: 5’-CTAAGAATTTCACCTCTGACAGT-3’ (reverse) [16] primers were used for amplification of a 207 bp fragment of the 18S rRNA gene of the *B. motasi*. In each nested reaction 1.7 μl of the primary PCR product was used as the template in a total volume of 14 μl containing 10 × PCR buffer 1.4 µl, MgCl_2_ 2.5 mM, dNTP 0.2 mM, inner primers 0.2 pM of each (Bioneer Inc., Korea), Taq DNA polymerase 1.4 U. The thermal condition for this stage was the same as described above. Finally, gel electrophoresis was performed on the PCR products using 1.5% agarose gel containing 15 μl/dl Safe stain (CinnaGen Co., Iran).

The PCR products with the expected size of both outer and inner segments were sequenced in forward and reverse directions (ABI 3130xl Genetic Analyzer, USA; Pouya Gostar Zhen Co., Iran). The validity of the obtained sequences was confirmed using nBLAST program and alignment to the corresponding sequences available in the GenBank.

### Statistical analysis

Statistical analysis was performed using SPSS software (version 21.0, SPSS Inc.). The Chi-square test was used to assess the relationship between parasitic infection (presence of *B. ovis* or *B. motasi*) and age (<1 year old and >1 year old), gender (female and male), and species of animals (sheep and goats). p*<*0.05 was considered significant.

## Results

**Figure-2:**
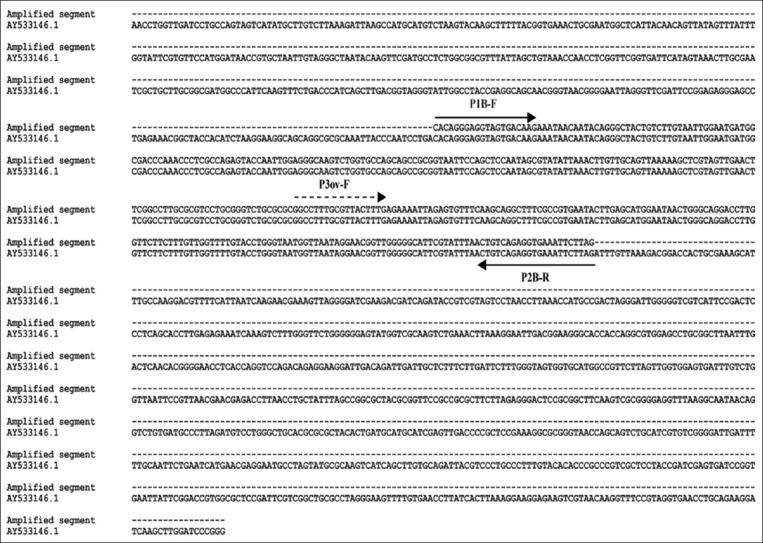
Alignment of amplified sequences to the *Babesia ovis* 18S ribosomal RNA gene. Amplified target sequences are shown between two pairs of the corresponding primers. The amplified sequences were completely identical to the GenBank report (GenBank: AY260178.1).

The nBLAST analysis and comparison of the PCR products sequences with the *B. ovis* 18S small subunit ribosomal RNA gene sequence (GenBank: AY533146.1) demonstrated that the sequences were identical to the GenBank report (Figure-2).

**Figure-3:**
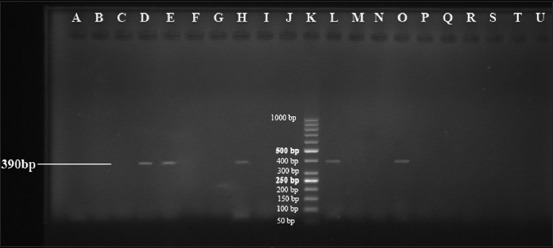
Primary polymerase chain reaction products were electrophoresed on 1.5% agarose gel to demonstrate a 390-442 bp fragment of 18S rRNA gene of *Babesia* and *Theileria*. Positive control: L; Negative control: M; Positive samples: D, E, H, O; Negative samples: A, B, C, F, G, I, J, N, P, Q, R, S, T, U; Ladder 50 bp: K.

As shown in Figure-3, the PCR with the primers (P1B-F/P2B-R) indicated that 19 (11.44%) out of 166 blood samples were infected to *Babesia* and *Theileria* spp. of which 16 (9.64%) and 3 (1.8%) samples were related to sheep and goats, respectively.

**Figure-4:**
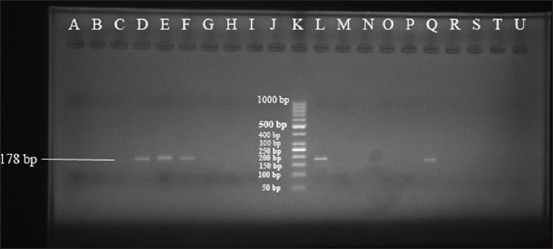
Semi-nested polymerase chain reaction products were electrophoresed on 1.5% agarose gel to demonstrate a 178 bp fragment of 18S rRNA gene of *Babesia ovis* using specific primers. Positive control: L; Negative control: M; Positive samples: D, E, F, G, Q; Negative samples: A, B, C, H, I, J, N, O, P, R, S, T, U; Ladder 50 bp: K.

Semi-nested PCR with specific primers of *B. ovis* (P3ov-F/P2B-R) showed that among 166 PCR products of the first stage, 38 (22.89%) samples were positive for *B. ovis* infection (Figure-4).

It can be seen from the data in Table-1 that there was no significant difference in the infection rate of *B. ovis* between the animals less and more than 1-year-old.

**Table-1:** The relationship between *B. ovis* infection and age, gender, and species of animals.

Results	Variables					

	Age		Gender		Species	
		
	<1 year	>1	Female	Male	Sheep	Goat
Positive (%)	12 (18.5)	26 (25.7)	32 (21.9)	6 (30)	29 (23.6)	9 (20.9)
Negative (%)	53 (81.5)	75 (74.3)	114 (78.1)	14 (70)	94 (73.4)	34 (79.1)
Total	65	101	146	20	123	43
p value	p>0.05		p>0.05		p>0.05	

Furthermore, no significant relationship was observed between the *B. ovis* infection and gender of animals.

The number of infected sheep 29 (17.5%) to *B. ovis* was more than the infected goats 9 (5.4%). However, no significant differences were found between the species of animals and the infection rate of *B. ovis* (Table-1).

**Figure-5:**
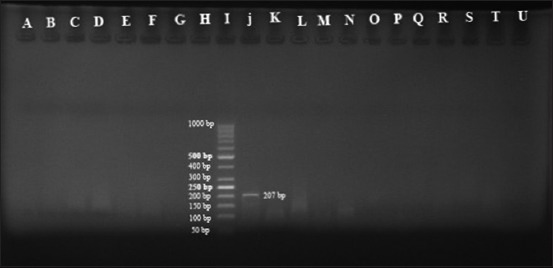
Semi-nested polymerase chain reaction products were electrophoresed on 1.5% agarose gel to demonstrate a 207 bp fragment of 18S rRNA gene of Babesia motasi using specific primers. Positive control: J; Negative control: K; Negative samples: A, B, C, D, E, F, G, H, L, M, N, O, P, Q, R, S, T, U; Ladder 50 bp: I.

In addition, semi-nested PCR with specific primers of *B. motasi* (P4mot-F/P2B-R) was accomplished on the PCR products, but none of these samples exhibited any visible band for *B. motasi* on agarose gel electrophoresis (Figure-5).

The number of positive and negative cases in PCR and semi-nested PCR for *B. ovis* are shown in Table-2. Out of 38 positive samples in the semi-nested PCR of *B. ovis*, only 13 samples were also positive in the first PCR. Due to the low parasitemia, the remaining positive samples of semi-nested PCR (25 samples) did not produce a visible band in the first PCR.

**Table-2:** The number of positive and negative cases in PCR and semi-nested PCR of *B. ovis*.

PCR Results	PCR+	Semi-nested PCR of *B. ovis+*
PCR+	19	13
PCR−	0	25
Seminested PCR of *B. ovis*+	13	38
Seminested PCR of *B. ovis*−	6	0

*B. ovis=Babesia ovis*, PCR=Polymerase chain reaction

Interestingly, among 19 positive samples of the first PCR, 6 samples did not show a visible band both in the semi-nested PCR of *B. ovis* and *B. motasi*.

## Discussion

The most common technique for diagnosing of clinical babesiosis in small ruminants is Giemsa staining [17]. However, this method does not have good sensitivity and specificity for detecting different species of *Babesia*. In addition, it is not an appropriate method for identifying of subclinical babesiosis in sheep and goats [8]. Molecular methods can be a good alternative for detection of these conditions. In this regard, the traditional PCR technique was frequently used in the epidemiologic studies of piroplasmosis in small ruminants [7,8,11]. However, this method cannot differentiate the various species of *Babesia* and *Theileria*; so, another molecular technique such as semi-nested PCR or restriction fragment length polymorphism is needed for this purpose. Therefore, the present study was conducted to determine the frequency of *B. ovis* and *B. motasi* by semi-nested PCR in the blood samples of small ruminants in East Azerbaijan Province, Iran.

In the first PCR, 19 animals were found to be infected with *Babesia* or *Theileria* genus. Through the semi-nested PCR, the number of infection to *B. ovis* was 38 animals, although *B. motasi* infection was not detected in any of the examined animals. It is interesting to note that 6 positive samples of the first PCR did not show any visible band both in the semi-nested PCR for *B. ovis* and *B. motasi*. As a result, the latter positive samples may belong to another species of *Babesia* or *Theileria*.

In accordance with our results, most of the molecular studies demonstrated that *B. ovis* was the main causative agent of ovine babesiosis in Iran [5,11]. However, one case of *B. motasi* infection among 220 sheep and goats has been reported in a molecular study in Iran [13]. As well as molecular studies in the neighbor countries of Iran such as Turkey [6,7,16,18] and Pakistan [19,20] indicated that *B. ovis* was the main causative agent of ovine babesiosis which were in agreement with our results.

The current study found that the infection rate of *B. ovis* among small ruminants, sheep, and goats was 22.89%, 23.6%, and 20.9%, respectively. Several studies have been conducted in different regions of Iran to detect the frequency of *Babesia* infection among sheep and goat populations. For example, in a biomorphometrical and molecular study, the frequency of *B. ovis* among 154 sheep was 5.85% [5]. Furthermore, the infection rate of *B. ovis* in the small ruminants of East Azerbaijan, West Azerbaijan, and Razavi Khorasan provinces was 14%, 16.7%, and 0.99%, respectively [8,21,22]. In accordance to our findings, a systematic review and meta-analysis on the status of babesiosis in Iran demonstrated that the prevalence of babesiosis in sheep and goats was 21.8% and 10.4%, respectively [12]. Although it is difficult to compare the results of this and prior studies due to different sample size and various techniques used to identify *B. ovis* infection, the previous investigations indicate that ovine babesiosis is endemic in different regions of Iran.

The results of this study indicate that the number of infected animals with more than 1-year-old was greater than the animals with <1 year old. However, there was no significant difference between age and *B. ovis* infection. The present findings seem to be consistent with other researches which found no significant relationship between the age of the animal and infection with *B. ovis* [4,6,19]. In addition, no significant association was observed between the *B. ovis* infection and gender of animals. This finding is in agreement with the findings of Aktaş *et al*. [6], which showed that there was no significant relationship between gender and *B. ovis* infection. However, in contrast to our finding, Iqbal *et al*. [19] exhibited that the association between gender and *B. ovis* infection was statistically significant. A possible explanation for this might be that due to the increase of infected ticks in warm season in this area, all age groups and gender of sheep and goats are constantly subjected to tick bites; so, the state of enzootic stability has been established, as described by others [6,17,23].

Results of the present study showed that the number of infected sheep more than infected goats. However, no significant difference was found between the species of animals and infection to *B. ovis*. In contrast to our results, some studies demonstrated that the susceptibility of sheep to *B. ovis* infection were more than goats [4,8,19]. It has been described that in the field condition, sheep were more susceptible to clinical disease caused by *B. ovis* infection than goats [24].

## Conclusion

The present study set out to determine the prevalence of *Babesia* infection in sheep and goats in Northwest of Iran by the semi-nested PCR technique. The study has shown that the semi-nested PCR is a precise method for differentiation of *Babesia* spp. The results of this investigation indicate that *Babesia* infection is highly prevalent (22.89%) among sheep and goat populations in East Azerbaijan Province, Northwest of Iran. In this study, we found no evidence for *B. motasi* infection. Therefore, it seems that *B. ovis* is the only species caused ovine Babesiosis in this region. The current study did not evaluate the tick vectors of ovine Babesiosis. Hence, further investigations on the tick vectors of babesiosis are needed to provide better control strategies for the disease.

## Authors’ Contributions

AB planned and carried out the study, AA carried out the study. AI planned, designed, and supervised the experiment. HA participated in the collecting data, analysis, and interpretation of data and drafting of the manuscript. All authors read and approved the final manuscript.
